# The Essential Need for a Validated Potency Assay for Cell-Based Therapies in Cardiac Regenerative and Reparative Medicine. A Practical Approach to Test Development

**DOI:** 10.1007/s12015-021-10244-5

**Published:** 2021-08-31

**Authors:** Lidia Gómez-Cid, Lilian Grigorian-Shamagian, Ricardo Sanz-Ruiz, Ana S. de la Nava, Ana I. Fernández, María Eugenia Fernández-Santos, Francisco Fernández-Avilés

**Affiliations:** 1 Department of Cardiology, Hospital General Universitario Gregorio Marañón, 28009 Madrid, Spain; 2 Instituto de Investigación Sanitaria Gregorio Marañón, Hospital Gregorio Marañón, 28009 Madrid, Spain; 3 CIBERCV, ISCIII, 28029 Madrid, Spain; 4 Departamento de Bioingeniería e Ingeniería Aeroespacial, Universidad Carlos III de Madrid, 28911 Leganés, Spain; 5 Faculty of Medicine, Universidad Complutense, 28040 Madrid, Spain; 6 ITACA Institute, Universitat Politècnica de València, 46022 Valencia, Spain

**Keywords:** Cell therapy, Potency test, Mechanism of action, Cardiac regenerative and reparative medicine

## Abstract

Biological treatments are one of the medical breakthroughs in the twenty-first century. The initial enthusiasm pushed the field towards indiscriminatory use of cell therapy regardless of the pathophysiological particularities of underlying conditions. In the reparative and regenerative cardiovascular field, the results of the over two decades of research in cell-based therapies, although promising still could not be translated into clinical scenario. Now, when we identified possible deficiencies and try to rebuild its foundations rigorously on scientific evidence, development of potency assays for the potential therapeutic product is one of the steps which will bring our goal of clinical translation closer. Although, highly challenging, the potency tests for cell products are considered as a priority by the regulatory agencies. In this paper we describe the main characteristics and challenges for a cell therapy potency test focusing on the cardiovascular field. Moreover, we discuss different steps and types of assays that should be taken into consideration for an eventual potency test development by tying together two fundamental concepts: target disease and expected mechanism of action.

**Graphical Abstract:**

Development of potency assays for cell-based products consists in understanding the pathophysiology of the disease, identifying potential mechanisms of action (MoA) to counteract it and finding the most suitable cell-based product that exhibits these MoA. When applied, the potency assay needs to correlate bioactivity of the product, via a measurement related to the MoA, with treatment efficacy. However, in the cardiovascular field, the process faces several challenges and high requirements.

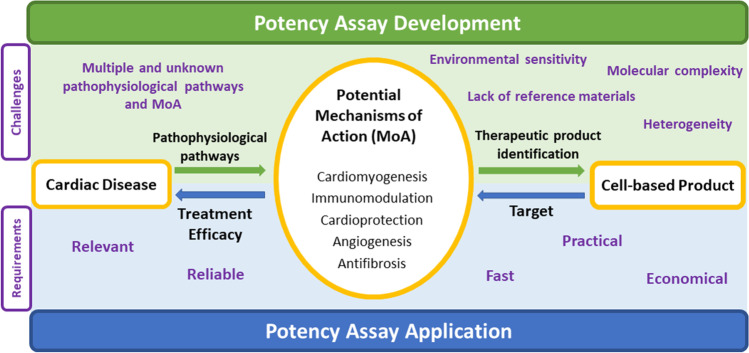

## Introduction

Medical landscape is being dramatically changed by the introduction of cell-based therapies [1]. In the cardiovascular field, research in regenerative and reparative medicine made an enormous contribution to the understanding of profound pathophysiological processes underlying different diseases and biomolecular cellular organization and functioning. However, in clinical trials of the field, cell-based therapies have only shown modest efficacy. This has been due in part to a lack of standardization, of comparability studies and of deep understanding of the mechanisms of actions (MoA) behind [2].

Development of adequate potency assays that reproducibly measure the ability of the cell-based ingredient to produce a given result (bioactivity), will be a step forward not only in industry, but also to overcome these limitations in academic research [3]. In fact, the International Council for Harmonisation of Technical Requirements for Pharmaceuticals for Human Use (ICH) recommends consideration of biological activity, alongside identity and purity among others, for determining product acceptance before intended use [4]. However, differently from small-molecule drugs (long dominating the pharmaceutic industry), cell-based therapeutics present higher inherent complexity and presumable several MoA that require a longer time to attain the clinical scenario and that complicate the development of adequate potency measurements. Key aspects are identifying critical quality attributes (CQAs) related to the MoA of the product, how to determine their acceptable ranges, and how to measure them reproducibly and feasibly on a routine basis.

In this article, we review the need and the requirements in developing potency assays for cell-based therapeutics in the cardiovascular field focusing on the recommendations by the ICH and those adopted by the U.S Food and Drug Administration (FDA) and the European Medicines Agency (EMA). Moreover, we discuss the specific challenges and the practical limitations of potency tests in the cardiovascular field not sufficiently commented in the regulatory papers. In addition to that, we elaborate on the link between the target cardiovascular disease and the expected MoA of the cell-based ingredient to propose potential candidates as in vivo, in vitro and surrogate measurements to evaluate potency of the product.

## Potency Assay as Regulatory Recommendation to Optimize Research Results

Potency has been defined as a quantitative measure of biological activity [4]. It can be considered equivalent to strength and defined as the therapeutic activity of the drug product [5, 6]. Therefore, a potency assay is a test or set of tests with the ability to confirm that the relevant biologic functions that correlate to the efficacy are present in the active ingredient and in the final product [7].

Product consistency is one of the pillars for the reproducibility of clinical effects and demonstrated efficacy. Identity, purity, stability, and quality, together with potency, need to be guaranteed to ensure this consistency. Therefore, validated potency assays are necessary before product release to support consistency in the strength of all released products. In fact, both the EMA [8] and the FDA [6] have pointed the need for measuring biological activity via a validated potency assay for qualification, validation and control of cell-based therapies [9]. In this regard, data demonstrating that the potency assay(s) measures an appropriate biological activity of the tested therapeutic agent is preferred when the material is ready for the first clinical trial [5].

However, cell-based therapies present several differences with respect to many classical pharmacological compounds (small molecular drugs). As “living products”, cells present larger variability, more limited stability, and larger molecular and mechanistic complexity. Their biological activity and therefore efficacy will strongly rely on their source, processing and/or storage. Viable cells may lose their biologic function during processing or storage [7], or change their properties in response to their environment with potentially significant functional and safety consequences [10]. As a result, merely confirming cell identity and viable cell number at the moment of product release does not necessarily correlate to biological activity measurements [8].

Solid potency tests are not only useful for ensuring that the final released product is consistent, effective, and high-quality manufactured. They also serve during the different stages of product development and manufacturing as part of product comparability, stability testing and quality evaluation [6, 8]. Adequate potency tests will allow the fair comparison of different products (different batches, different cell types or even different sources), and therefore the selection of the optimal one regarding a specific MoA. In addition to that, the potency assay may also be useful to assess stability by ensuring the strength of the product is maintained at the different stages of the manufacturing process and determining product shelf-life and optimum manufacturing and storage conditions. Investment in a solid potency assay from early stages of product development mitigates the risk of costly product failure in subsequent states [7].

## Requirements of an “Ideal” Potency Assay

An ideal potency assay should be relevant, practical, reliable and ideally quantify the biological activity related to the MoA [6, 8]. Although tolerant with the heterogeneity of the product, the assay must be able to detect meaningful changes potentially related to efficacy [6] and have predefined acceptance and rejection criteria [7] to conclude if the product is suitable for release, as well as its suitable dating periods. To be reliable, potency tests should have adequate reference materials: standards and/or controls to ensure interoperator and time-to-time consistency. Also, tests should report on accuracy, sensitivity, specificity and reproducibility to be amenable for validation and meet labelling requirements [6].

**Fig. 1:**
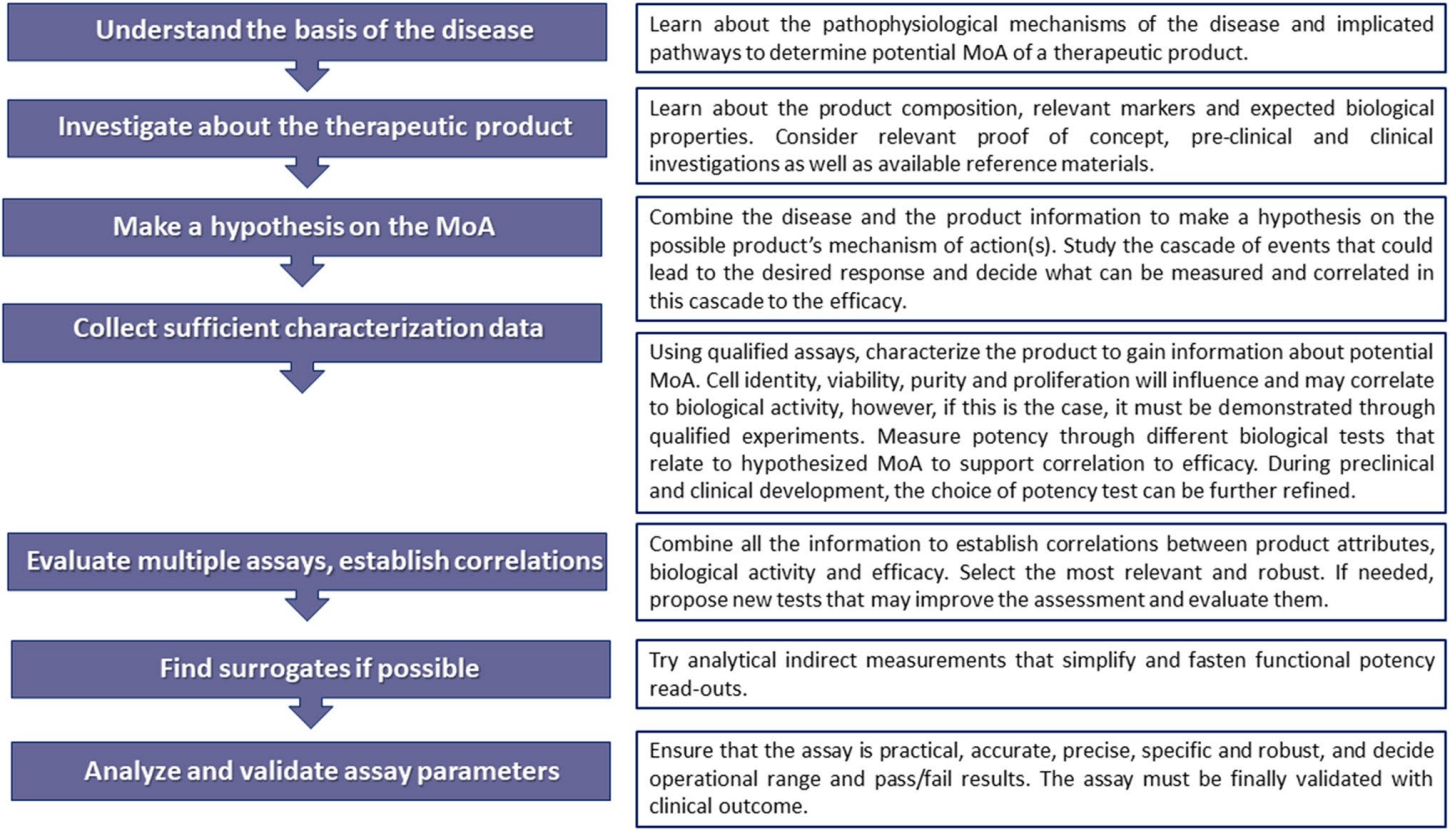
Steps in the development of a potency assay

Figure 1 summarizes the steps to be followed in the process of a potency test development. When characterizing the product identity, it is important to appropriately control the mixture composition if several cell types are present in the product. Moreover, while identity, cell purity and proliferation will influence and may correlate to biological activity and potency, this must be demonstrated through qualified experiments.

## Challenges in the Development of Potency Assays for Cell-Based Therapies

Development of a potency assay for cell-based products faces several challenges. One is the heterogeneity of the primary cells partially related with the particularities of their donors and the intention of use as an autologous or allogeneic treatment [6]. Allogeneic treatments have presented similar effect but several advantages over autologous treatments, such as larger availability and the possibility to select the most potent products [11]. Other factors such as cell age and their previous exposure to risk factors can also determine potency and are more easily controlled in allogenic treatments. In cell-based products, and in particular in autologous treatments, tests requiring minimal amount would be preferred as the availability is considerably limited.

In addition, cell-based products present a high level of molecular complexity [8, 12], as well as multiple, and usually not fully-known MoA [13], entangling the selection of the most accurate potency test. Thus, as one single test may not be sufficient to fully represent several MoAs of the product, the final potency assay may be based on multiple complementary tests, the so called assay matrix [14]. Moreover, when cell-based product is administered in vivo, the biodistribution, as well as the retained amount of product in the target tissue are usually crucial for the efficacy but can be complex to model. This can considerably difficult establishing links between in vitro potency assays and the physiological mechanism of action in vivo [15].

Cells are also very sensitive to environmental conditions, processing, storing, delivery, and administration, what affects their viability and quality attributes, and therefore, their stability and shelf-life [8]. So, despite potency should be measured on the final product, stability (referring to maintenance of its physiological and potency attributes in time) should also be demonstrated during the different phases of product’s life. After the stability is confirmed at all the stages, potency measured at a given time-point can be used as indicative of product’s quality [16].

Another important limitation is the lack of adequate reference materials and standards. To guarantee consistency, reference materials and standards must be highly characterized and be sufficiently homogenous and stable [17]. However, when attempting to quantify CQAs in biological materials, standardization becomes difficult as the tests commonly lack an existing certified reference material to use for comparison and often do not provide measurements in independent and internationally recognized units (for example, in International System of Units) [18]. The heterogeneity and complexity of living cells difficult the development of a reference cell line [15] or a “cell-ruler” [19] to normalize the potency measurement over several batches.

Determining acceptance and rejection criteria in cell-based products potency assays is not always trivial. The acceptance criteria may be redefined as new knowledge about the product is gained in vitro, in vivo, and in the clinical scenario during the different stages of product and potency assay development. It is necessary to ensure that the final defined acceptance criteria consists in a numerical range that ensures biological activity and reflects clinical effectiveness [6].

The bioactivity of the product can be evaluated in vitro and/or in vivo [5]. Potency assays performed in vivo should include carefully defined controls, including sham and vehicle treatment groups, and take into consideration specific xenogeneic responses that might influence the induced effects. Main drawbacks of in vivo models for routine potency assessment are the costs and the time consumption [16]. Therefore, in vitro models are generally preferred [20]. In vivo testing is commonly used to validate the in vitro predictability and to identify the pass/fail results for potency, as in vitro tests should correlate to the intended effect in vivo and assumed MoA.

In vitro functional assays, such as endothelial tube formation for angiogenesis or anti-inflammatory assessment are often not suitable for routine testing right before the product is administered since they present high variability and are time-consuming [8]. Therefore, when possible, the use of surrogate (non-direct) measurements that have previously demonstrated correlation with functional assays, such as for example specific gene expression or secretion of a factor, are preferred [5, 6].

## Disease-Targeted and Mechanism of Action-Guided Potency Tests in Cardiovascular Regenerative Medicine

Cell therapy products have been indistinguishably used for a wide range of cardiac diseases over the past two decades many times with just modest efficacy [1, 21]. However, the role of the pathophysiological pathways underlying each of the diseases should not be underestimated in the therapeutic response of a given cellular product and should be taken into consideration for the right selection of the potency test. While a specific cell-product, with predominantly one MoA can be suitable for a particular condition, it may be inappropriate for a different disease. Moreover, the pathophysiological stage of the disease, acute or chronic, and the predominantly inflammatory or fibrotic underlying background, are also important factors to consider when opting for a particular product. Products with more immunomodulatory, anti-inflammatory and cardioprotective (anti-apoptotic) mechanisms of action may be preferred in early stages of the disease, while proangiogenic, anti-fibrotic and strategies targeting direct re-muscularization may be more beneficial in advanced stages of the disease.

**Fig. 2:**
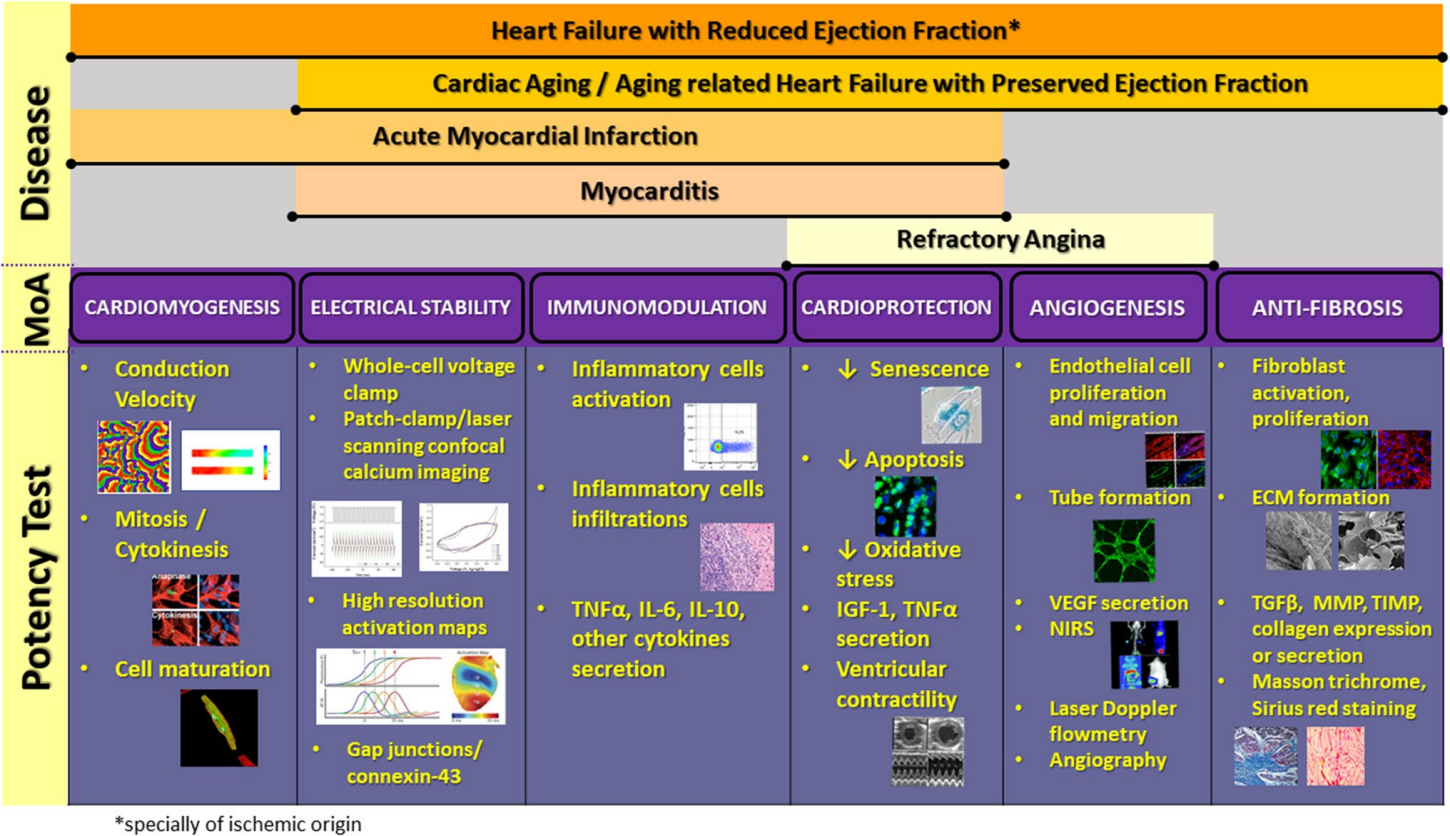
Potency tests based on the expected mechanism of action (MoA) for different cardiac diseases

Several MoA linked to cell-based therapies in cardiovascular regenerative medicine have been described [22]: cell survival and protection (cardioprotection), immunomodulatory, anti-inflammatory and anti-fibrotic effects, angiogenic and cardiomyogenic [23, 24]. To develop an adequate potency assay and successfully quantify the potential efficacy of a cell-based product, it is essential to identify the most relevant MoA of the therapeutic product and at least one of the disease-related significant pathophysiological pathways expected to be counteract by the former (Fig. 2).

Some cell products have been characterized for their MoA, but a robust quantification of these effects to compare and identify the most potent cell type for a certain clinical condition, and/or from batch to batch is still lacking [2]. Today is accepted that the cells exert their therapeutic effects mostly through paracrine secretion of soluble factors and/or extracellular vesicles [16, 19, 25, 26]. Indeed, extracellular vesicles offer the potential to overcome the frailty of cell therapy by conserving their bioactivity regardless of the extremes of handling [27]. Moreover, the use of extracellular vesicles, exosomes, or other cell-derived products ease standardization, costs and are more manageable to scale-up [21].

When the expected MoA is paracrine-mediated, the potency assay should initially focus on detection of secreted bioactive molecules or particles related to the process, such as cytokines, growth factors, miRNA, etc. and then, if possible, find surrogate markers [8]. These analytical assays can evaluate immunochemical, biochemical and/or molecular attributes such as cell surface markers, secretion factors, protein or gene expression patterns [20]. In case the intended effect is through tissue replacement (i.e. using pluripotent stem cells), then cell retention, differentiation potential and maturity of the differentiated cardiomyocytes should be considered for the potency assay.

## Possible Ways of Measuring Potency According to the Expected Mechanism of Action

Once the potential MoA linked to the cell-based therapy product and the target cardiovascular pathology have been identified, it is important to determine the potential tests that could be used to quantify potency. In this section, different in vivo, in vitro and surrogate tests for the different potential MoA (cardiomyogenesis, electromechanical coupling, immunomodulation, cardioprotection, angiogenesis and anti-fibrosis) are reviewed (Fig. 2).

### Cardiomyogenesis

Cardiomyogenesis refers to new cardiomyocyte formation that is now acknowledged to be rare in an adult human heart and still inefficiently achieved by different cell therapy modalities [28]. The newly formed cardiomyocytes can result from direct differentiation of the implanted cells, from differentiation of endogenous cardiac stem cells, or from proliferation of pre-existing cardiomyocytes. Cardiomyogenesis resulting from cardiomyocyte division can be confirmed ex vivo by using classical mitotic markers, such as ki-67, phospho-histone 3 and thymidine analogs such as bromo-deoxyuridine in multinucleated cardiomyocytes. However, these are not always indicative of true mitosis or cell division and require animals, which may difficult its use for routine testing. If still this is considered as the MoA in a given condition, the combined use of different mitosis and cytokinesis markers (e.g. Aurora B kinase) could increase the efficiency of the potency test measured. Cardiomyogenesis arising from implanted cell differentiation can be assessed in vitro by quantifying their potential to differentiate into the myocyte lineage under specific conditions. Nonetheless, the potential to differentiate in vitro may differ in vivo, so adequate correlation between the in vitro differentiation, in vivo differentiation and in vivo efficacy must be previously demonstrated.

### Electromechanical Maturation and Coupling

Exogenous or extrinsic replacement generally refers to implantation of cardiomyocytes differentiated in vitro from embryonic stem cells or induced pluripotent stem cells (iPSCs). In these studies, electromechanical integration and maturity of transplanted cells into the injured heart is fundamental to achieve functional improvement [29] and should be considered for a potency assay. To evaluate electrophysiological maturity and avoid potential pro-arrhythmic effects [30], the final construct should be assessed for activation frequency, conduction velocity and action potential duration and morphology to be compared to native tissue electrophysiological characteristics. Electrophysiological studies are also crucial when the pursued target of the therapeutic product is the generation of biological pacemakers or an antiarrhythmic effect [31, 32].

In vitro cell automaticity (spontaneous activation) can be studied using whole-cell voltage clamp and simultaneous patch-clamp/laser scanning confocal calcium imaging. High resolution activation maps that characterize impulse initiation and propagation can reveal important information in vitro about the electrophysiology of the cells to be implanted and about temporal coupling between graft and host cells ex vivo. However, these tests are not practical for routine and massive testing because they are usually labor intensive and require highly specialized and costly equipment. Therefore, despite up to date there are no alternative or validated measurements on this regard, it is recommended to explore and attempt to validate alternative in vitro or surrogate markers for electrophysiological stability and maturity. For example, for functional integration to occur, the maturity of the electrical potential generated by differentiated cells is dependent on the amount and proportion of ion channels and the ability of the electrical potential to propagate to neighbouring cells through gap junctions. Using protein and/or gene expression of ion channels and connexin-43 [33] in differentiated cardiomyocytes, if validated, could be indicative of electrical maturity of the product. Evaluating modifications in these proteins in vitro in models of target cells or tissues under the presence of the therapeutic product could be indicative of electromechanical coupling and antiarrhythmic potential of the therapeutic agent.

### Immunomodulation

Contrary to classical belief, inflammation is not necessarily an impediment to tissue regeneration. Some immune cells (monocytes and macrophages) are required for cardiac regeneration, and injury-induced cardiomyocyte proliferation is inhibited by immunosuppression [23]. Harnessing immune cells pro-reparative mechanism(s) to promote heart regeneration vs. their pro-inflammatory effect that exacerbates disease will still require better understanding. In fact, immunomodulation is gaining support as the main mechanism of action behind cell therapies in the cardiovascular field [34]. Despite the challenges in establishing the mechanisms behind immodulation, MSCs and CPCs have shown to attenuate production of TNF-α and IL-6, to increase the expression of IL-10 [35], and to polarize an effector macrophage population [36]. Regarding potency assays, cell therapy effect on inflammatory response can be assessed in vitro by measuring macrophage activation and migration, and secretion of pro- and anti-inflammatory factors, such as TNF-α, IL-6, and IL-10.

### Cardioprotection

Cardioprotection in terms of increased cellular resistance to internal or external stressors, is essential in limiting cardiac remodelling after injury, and therefore preserving cardiac function at a longer term. Cardiomyocyte loss is probably the main issue related with the disease progression in ischemic heart disease and/or systolic heart failure. These cells can be protected directly by means of antisenescence, antioxidative or antiapoptotic effects conferred by some therapeutic cells, or by stimulation of autophagy, among others [37]. Both MSCs and CPCs have shown to enhance cell survival, prevent apoptosis in cardiomyocytes and prevent ischemic injury via paracrine mechanisms [38–40].

Ventricular contractility (i.e. left ventricular ejection fraction) assessed by imaging techniques in vivo animals is accepted to translate underlying cardiomyocytes survival and function. They can also contribute to the elucidation of the particular mechanisms and to identification of new potential markers. However, animal studies are more expensive and non-efficient as a frequent potency assay for batch release. Alternatively, if possible, in vitro functional tests once demonstrated to be correlated to in vivo expected efficacy could serve to evaluate and quantify cardioprotective efficacy of the therapeutic product. In this regard, evaluation of the effect of the cell-based products on cardiomyocyte apoptosis and/or senescence could be considered as potential in vitro potency assessments for validation. As example of further surrogate potency endpoints, the measurement of the secretion or expression of some cytokines (i.e. IGF-1, TNF-α [41, 42]) that have demonstrated to be associated with the inhibition of apoptosis and increased cardiomyocytes survival could be considered.

### Angiogenesis

A continuous supply of nutrients, as well as routes for eliminating metabolic products, is essential for tissue health. In the absence of neovascularization following injury, the heart fails to repair and instead forms extensive fibrotic scar. Although the precise mechanisms of neovascularization are not well defined, endothelial cells proliferation and arrangement into tube-like structure finally leads to the formation of new vessels. Paracrine activity of MSCs and CPCs have shown to augment the pro-angiogenic activity of endothelial progenitor cells [43–45]. This effect has been linked to vascular endothelial growth factor (VEGF) secretion [43] and extracellular matrix metalloproteinase inducer (EMMPRIN) release.

In animal models, angiogenesis can be evaluated with angiography, laser Doppler flowmetry, near-infrared spectroscopy (NIRS), histology and immunohistochemistry [16]. In vitro proangiogenic potency can be analysed by the ability of the therapeutic agent to induce endothelial cell proliferation and migration, or tube formation on Matrigel. However, due to the inherent variability of Matrigel, reference material should be used to ensure test reproducibility. Synthetic alternative materials presenting higher sensitivity and reproducibility could be alternatively considered [46]. While secretion of involved factors, such as VEGF could serve as a potential surrogate measurement, analysing the combined secretion of different cytokines involved in angiogenesis instead of a single one, may increase the predictive value of the potency test [47].

### Anti-Fibrotic Mechanism

Another key mechanism of progression of different types of cardiac injuries is fibrosis, an increased extracellular collagenous deposition. Transforming growth factor beta (TGFβ) is the main player for inducing fibroblast activation and proliferation, extracellular matrix formation, and endothelial to fibroblast transdifferentiation [48]. Different cell-based products, such as MSCs and CPCs have demonstrated to possess an anti-fibrotic effect by transcriptional downregulation of types I and II collagen synthesis [49] and by driving fibroblast to a more therapeutic profile (higher stroma-cell-derived factor 1, SDF-1, and VEGF secretion) [50].

Fibrosis can be easily visualized and quantified ex vivo with Masson trichrome or Sirius red staining. However, in vitro fibrosis can be non-directly inferred for example by evaluating fibroblasts TGFβ, matrix metalloproteinase (MMP) or tissue inhibitor of metalloproteinases (TIMP) secretion under the effect of the therapeutic product. TGFβ, MMP and TIMP secretion by the therapeutic cell product itself could be also indicative of fibroblast recruitment potential and serve as a surrogate endpoint.

## Examples of Potency Assays for Cell-Based Therapy Products in the Cardiovascular Field

Among the most detailed potency assays for cell-based therapy products in the cardiovascular field are the one for Amorcyte, for MultiStem and for CardiAMP. Amorcyte, AMR001 is an autologous cell-therapy for the treatment of myocardial infarction based on CD34 + CXCR4 + cells. Several parameters were measured during phase I clinical, but only mobility in an SDF-1 gradient correlated to efficacy. Therefore, the MoA was thought to be product mobilization and migration to the damaged tissue along an SDF-1 gradient where administered cells facilitate tissue repair and vascular regeneration. As a result, an in vitro migration potency assay of CD34 + CXCR4 + cells in a defined SDF-1 gradient was used in later phases [7, 51]. MultiStem is another cell treatment for myocardial infarction among other pathologies, but that uses allogeneic bone-marrow derived stromal cells. The main MoA is thought to be angiogenesis induction. The initial potency assay determined the minimum levels of VEGF, CXCL5 and IL-8 secreted by the cells that led to adequate tube formation, and finally secretion of these factors were replaced by their gene expression levels as surrogate markers [7, 51]. Recently, BioCardia has patented a new potency assay for their CardiAMP product, which consists in autologous bone marrow cells (BMCs) for the treatment of chronic myocardial ischemia or heart failure of ischemic origin. The potency assay, based on previous clinical trial results, defines a minimum number and proportion of CD19 + , CD34 + and CD133 + cells in BMCs from the patient and before expansion to be related to efficacy [52]. Other potency assays and their corresponding validation have been investigated in the academy. One consists in a rapid cell invasion assay for identifying functional BMCs across Matrigel-coated transwells with an electric cell-substrate impedance sensing [53]. Other consists in an in vitro functional analysis for CPC-derived exosomes at GMP-Grade Manufacturing for evaluating their anti-apoptotic effect in CPC and HL-1 cardiomyocytes and their pro-angiogenic activity through tube formation and amount of CD31 expression in endothelial cells [54].

## Conclusion

The use of in vivo and some in vitro assays, although closer to the clinical scenario and more representative of the MoA, commonly present high costs and are time consuming, making them unfeasible for mass production. The use of simple and easier to scale assays such as surrogates related to the specific MoA (i.e. as gene and protein expression or the secretion of specific factors) could be more practical. For cardiomyogenesis, potential to induce cardiomyocyte division or to differentiate into the cardiomyocyte lineage is proposed. Expression of ion channels (sodium, calcium and potassium channels) and connexin 43 could be indicative of electromechanical maturation and coupling, whereas secretion of pro- and anti-inflammatory factors (TNF-α, IL-6 and IL-10) could determine immunomodulation. The antisenescent and antiapoptotic potential of the product can be determined in vitro on cardiomyocyte and stromal cells, VEGF and specific cytokine secretion can be indicative of the angiogenic potential, and TGFβ, MMP and TIMP secretion of the antifibrotic potential. However, to develop these, or other scalable and accurate potency assays, further work is needed to validate that the potential surrogate measurements correlate to the efficacy of the product in patients.

Development of a potency assay for cell-based therapeutics in the cardiac field is now considered a priority by the regulatory agencies to optimize product efficacy in patients. Despite the challenges related with reference materials and the complexity of the biological treatments, all attempts should be made to develop robust and reproducible potency assays from early stages of product development. The potency assays should adequately reflect the product’s relevant biological properties related to their expected MoA in the target cardiovascular disease.

## Data Availability

Not applicable.
